# Recent updates in click and computational chemistry for drug discovery and development

**DOI:** 10.3389/fchem.2023.1114970

**Published:** 2023-02-07

**Authors:** Jiang Hong Cai, Xuan Zhe Zhu, Peng Yue Guo, Peter Rose, Xiao Tong Liu, Xia Liu, Yi Zhun Zhu

**Affiliations:** ^1^ State Key Laboratory of Quality Research in Chinese Medicine, School of Pharmacy, Macau University of Science and Technology, Taipa, Macau, China; ^2^ Department of Clinical Pharmacy, School of Pharmacy, Second Military University, Shanghai, China; ^3^ School of Biosciences, University of Nottingham, Nottingham, United Kingdom; ^4^ Shanghai Key Laboratory of Bioactive Small Molecules, Department of Pharmacology, School of Pharmacy, Fudan University, Shanghai, China

**Keywords:** click chemistry, computational chemistry, CADD, druggable candidates, drug development

## Abstract

Drug discovery is a costly and time-consuming process with a very high failure rate. Recently, click chemistry and computer-aided drug design (CADD) represent popular areas for new drug development. Herein, we summarized the recent updates in click and computational chemistry for drug discovery and development including clicking to effectively synthesize druggable candidates, synthesis and modification of natural products, targeted delivery systems, and computer-aided drug discovery for target identification, seeking out and optimizing lead compounds, ADMET prediction as well as compounds synthesis, hopefully, inspires new ideas for novel drug development in the future.

## Graphical Abstract

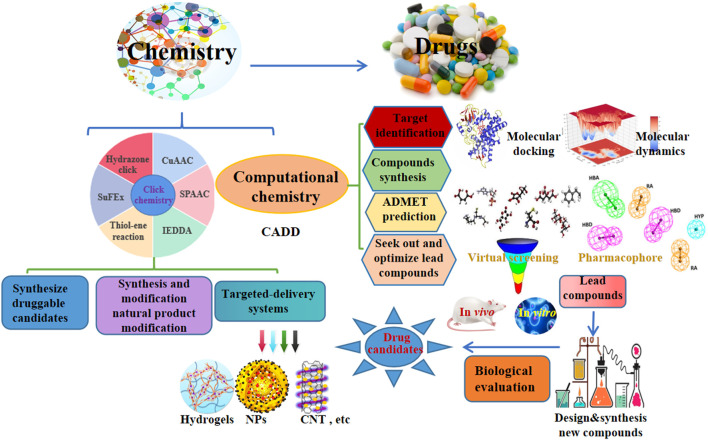

## Introduction

Click chemistry, an efficient chemo-selective synthesis method for coupling molecular fragments under mild reaction conditions, mainly includes Cu-catalyzed azide-alkyne cycloaddition reaction (CuAAC), strain-promoted azide-alkyne cycloaddition reaction (SPAAC), thiol-ene reaction, inverse electron demand Diels–Alder reaction (IEDDA), hydrazone click chemistry and the newly emerging sulfur fluoride exchange (SuFEx) reaction, has been a hot research topic in the field of chemistry since it was first reported in 2001 (Zhang et al., 2021a; Ashe, 2022). Computer-aided drug design (CADD) has attracted a lot of attention for its potential to accelerate and reduce the cost of the drug development process (Wu et al., 2020). In addition, natural products provide a variety of lead compounds and novel drugs, are worthy of further development. Furthermore, early and late-stage development of new drugs may be slowed down by problems such as poor target selectivity or side effects, toxicity, resistance, inappropriate physicochemical and pharmacokinetic properties. Therefore, we summarized the recent applications of click and computational chemistry in drug development such as click to effectively synthesize druggable candidates, synthesis and modification of natural products, targeted delivery systems including hydrogels, nanoparticles (NPs), carbon nanotubes (CNT), etc, and computer-aided drug discovery including molecular docking and molecular dynamics to identify target, virtual screening (VS.) and pharmacophore to found and optimize lead compounds, ADMET prediction as well as compounds synthesis, which are making a splash in new drug development, hopefully, providing new insights for the discovery of new drug from click and computational chemistry.

## Click chemistry

### Click to efficiently synthesize druggable candidates

**FIGURE 1:**
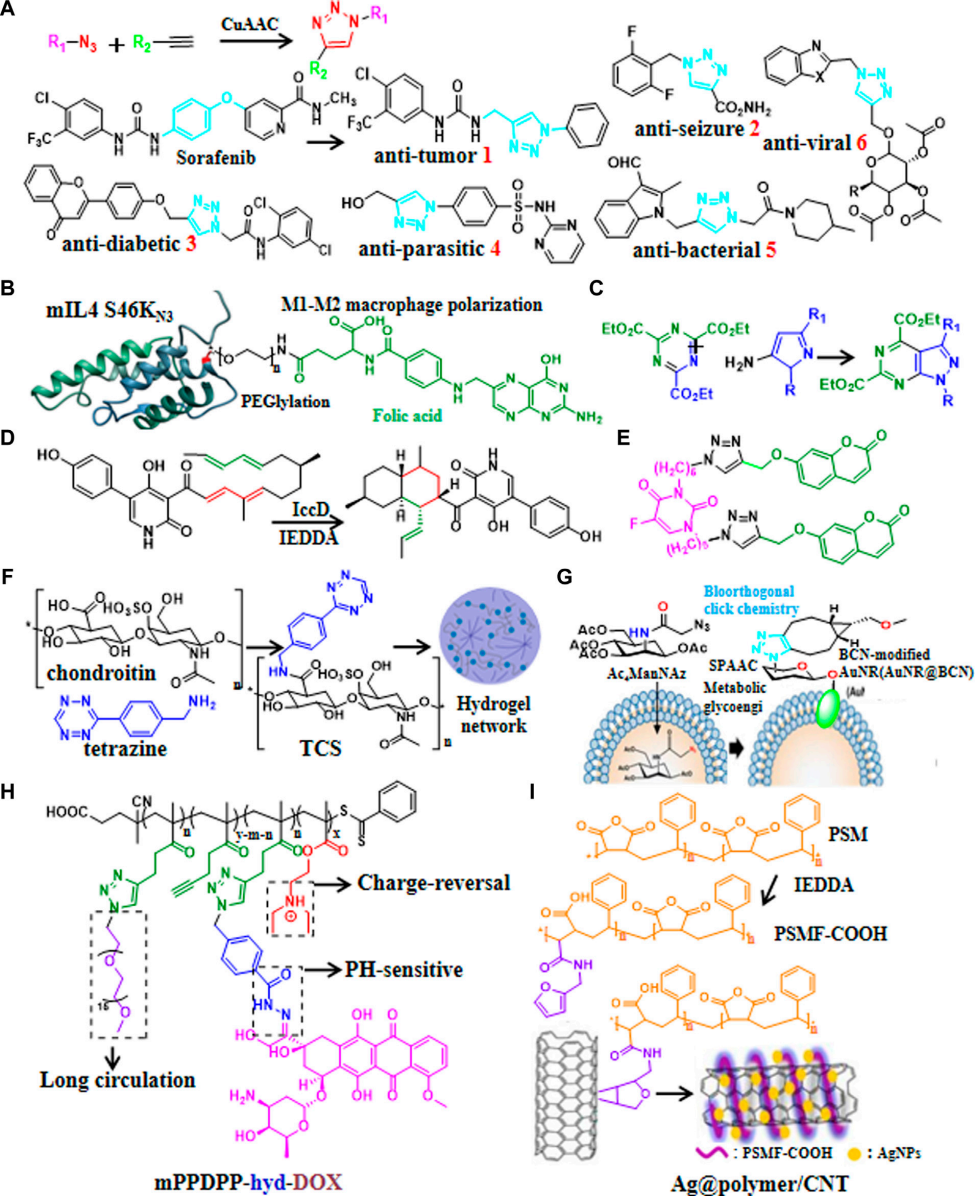
Recent updates in click chemistry for drug discovery and development. **(A)** Reaction formula of CuAAC and some recent applications of CuAAC for developing drug candidates containing 1,2,3-triazoles ring. **(B)** An example of natural product modification to improve half-life and target IL-4 to arthritic joint via SPAAC. **(C)** An example of introduction aromatic heterocycles via IEDDA. **(D)** An example of efficient synthesis of natural products via IEDDA. **(E)** An example of the generation of anti-cancer drug candidate by modification of the natural product coumarin via CuAAC. **(F)** Catalyst-free click chemistry to generate chondroitin sulfate-multiarmed PEG hydrogels for skin tissue engineering. **(G)** An example of the generation of MSCs-mediated deep tumor delivery of gold nanorod for anti-tumor therapy via SPAAC. **(H)** An example of polymer nanomicelle platform for cancer treatment via CuAAC. **(I)** An example of the generation of silver nanoparticle-supported polymer-encapsulated carbon nanotubes (CNTs) via IEDDA for nonenzymatic glucose sensing and antimicrobial activity applications.

The transformation of the active compound skeleton is a magic weapon for researchers to break through patent restrictions and improve the activity of compounds in the development of new drugs. Copper-catalyzed 1,3-dipolar cycloaddition (CuAAC) to form 1,2,3-triazoles is the most popular reaction in click chemistry. Recently, 1,2,3-triazole backbones with hydrogen bonds, moderate dipole moments and enhanced water solubility had been widely used to generate drug candidates of anti-tumor (Brown et al., 2022; Elganzory et al., 2022; Mohammed et al., 2022; Oekchuae et al., 2022; Oliveira et al., 2022; Mironov et al., 2023), anti-seizure (Bhattacherjee et al., 2022), anti-diabetic (Dhameja et al., 2022), anti-parasitic (Aljohani et al., 2022), anti-bacterial (Daher et al., 2022; Mokariya et al., 2022; Nsira et al., 2022) and anti-viral (Kutkat et al., 2022; Tatarinov et al., 2022) *via* CuAAC click chemistry (Figure 1A).

### Synthesis and modification of natural products

Natural products have provide abundant resources for drug discovery. Recently, click chemistry had been adopted for synthesis and modification of natural products, for instances, SPAAC was used to modularly generate Bcl-xL inhibitor (Brauer et al., 2022), adjust PEG chain length and targeting moiety to further improve half-life as well as targeting IL-4 to arthritic joint (Figure 1B) (Spieler et al., 2020). It was reported that poly (globalide-co-ε-caprolactone) could be functionalized with N-acetylcysteine side chains *via* thiol-ene reaction (Guindani et al., 2019). Furthermore, IEDDA could be used to introduce aromatic heterocycles (Figure 1C) (Xu et al., 2020) and triazines (Zhang et al., 2021b). Similarly, the synthetic efficiency of biosynthesis of anti-fungal drug candidate Ilicicolin H increased 3 × 10^5^ times *via* IEDDA (Figure 1D) (Zhang et al., 2019). Moreover, 5-fluorouracil-coumarin conjugation (Figure 1E) as anti-cancer drug candidate (ópez et al., 2022) and pH responsive doxorubicin delivery polymers nano-particles (Wallat et al., 2018)for treatment of breast and ovarian cancer were generated by modification of natural products *via* CuAAC. In addition, quercetin-gold quantum dots for adenocarcinoma treatment (Pansare et al., 2022) and chondroitin sulfate-multiarmed PEG hydrogels for skin tissue engineering (Sousa et al., 2022) had been developed by modification of natural products (Figure 1F).

### Targeted delivery systems

Existing drugs may have dis-advantages such as low selectivity, long synthetic routes, poor stability and side effects, thence the development of targeted delivery systems make great sense. Recently click-generated hydrogels had broad applications in the fields of anti-tumor (Ali et al., 2022; Bonardd et al., 2022), wound repair (Basurto et al., 2022) and long term regeneration therapy (Jang et al., 2021) *via* IEDDA, CuAAC,thiol-ene reaction, and SuFEx, respectively. Biomimetic stiffening of cell-laden hydrogels *via* sequential thiol-ene and hydrazone click reactions (Chang et al., 2021). Furthermore, nanoscale covalent organic frameworks (COFs) (Guan et al., 2022), Nisin-shelled nanoemulsion (Hashad et al., 2022), and MSCs-mediated deep tumor delivery of gold nanorod (Figure 1G) (Yun et al., 2022) had been synthesized for anti-tumor therapy *via* thiol-ene reactions, SPAAC, and SPAAC, respectively. Moreover, pH-sensitive polysaccharide-gold nanorod conjugate (Hou et al., 2019) and polymer nanomicelle platform (Figure 1H) (Liao et al., 2021) were reported to treat cancers *via* hydrazone click reaction and CuAAC, respectively. In addition, silver nanoparticle-supported polymer-wrapped carbon nanotubes (CNT) (Cao et al., 2022) for non-enzymatic glucose sensing and antimicrobial applications (Figure 1I), COF-based nanoreactors for click-activated pro-drug delivery and precise anti-vascular therapy (Wang et al., 2022) had been synthesized *via* IEDDA, these click chemistry-based targeting strategies may find widespread application in drug delivery in the future.

## Computational chemistry in drug discovery

**FIGURE 2:**
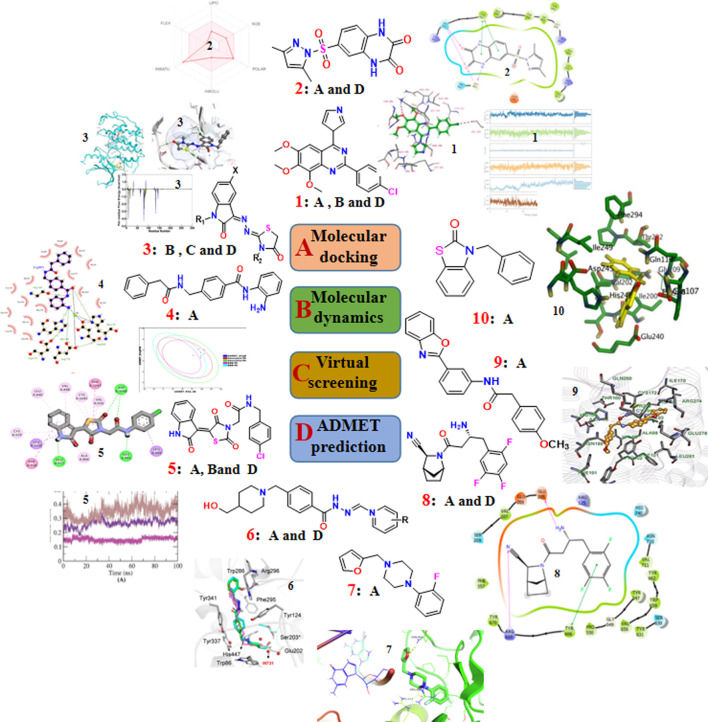
The recent updates of computational chemistry in target identification, lead compound discovery and ADMET prediction for drug development.

To effectively and efficiently design and develop new drugs, computational methods had been applied for drug design including target identification, seeking out and optimizing lead compounds prediction of pharmacokinetic and toxicological properties as well as compound synthesis by molecular docking and molecular dynamics, virtual screening, pharmacophore and ADMET prediction. Novel quinazoline derivative **1** as tubulin polymerization inhibitor (Dwivedi et al., 2022), PARP-1 inhibitor **2** (Syam et al., 2022), CDK2 inhibitor **3** (Qayed et al., 2022), HDAC-1-3 inhibitor **4** (Cheshmazar et al., 2022), VEGFR-2 inhibitor **5** (Taghour et al., 2022) were identified for cancer therapies. Furthermore, AChE inhibitor **6** (Macedo Vaz et al., 2022) for treatment of Alzheimer’s disease and Mtb RNAP inhibitor **7** (Mekonnen Sanka et al., 2022) for antitubercular and antimicrobial treatment were deserve further study. Moreover, a lead compound **8** of DDP4 inhibitor (Maslov et al., 2022) and acetamide derivative **9** (Zhou et al., 2022) as P2Y14R antagonist were considered as drug candidates for treating type 2 diabetes and gout, respectively. Additionally, potential SARS-CoV-2 main protease inhibitor **10** (Dong et al., 2023) and carbazole alkaloids from Murraya koenigii (Wadanambi et al., 2023) were identified as a promising drug candidates for inhibiting coronavirus infection. Surprisingly, it had been reported a computationally guided asymmetric total synthesis of resveratrol dimers, which possessed a wide range of biological activities such as antioxidant, anti-tumor and cardiovascular activities (Nakajima et al., 2022), suggesting that computationally guided organic synthesis may be a powerful strategy to advance the chemistry of natural products (Figure 2).

## Conclusion and prospects

In the review, we summarized recent updates in click chemistry for drug discovery and development, including chemical click synthesis of druggable candidates, synthesis and modification of natural products, targeted delivery systems. In addition, we introduced updated computational chemistry in drug discovery for target identification, discovery and optimization of lead compounds, compounds synthesis and prediction of pharmacokinetic and toxicological properties. Click chemistry is a very powerful tool in drug discovery, in which the synthesis of 1,2,3-triazole ring as a pharmacophore, bioisostere *via* CuAAC has great potential in the drug design for a variety of diseases, however, 1,2,3-triazole ring itself is not a commonly used pharmacophore, and it is rare in marketed drugs, indicating that the use of 1,2,3-triazole as drug molecules still has certain limitations. Furthermore, the CuAAC reaction introduces copper species into biological systems and organisms, leading to potential toxicity issues while many Cu chelation sites may inhibit catalyst activity. Moreover, Copper-free cycloaddition SPAAC reaction and IEDDA reaction have their own issues: for example, they are susceptible to side reactions with nucleophilic residues (e.g., thiol residues in glutathione), and the reactive (electrophilic) nature of the requisite cyclic alkynes/alkenes may result in poor regiospecificity. Although computer molecular docking and molecular dynamics have important applications for target identification, however, the protein used for molecular docking may have a huge unknown difference from the protein in the pathological state due to site mutation. Additionally, computational chemistry needs to be combined with more biological activity test and mechanism exploration. In a word, although click and computational chemistry have shortcomings, which still hold a great and unnegligible potential for drug discovery and development, hopefully, this review can stimulate new ideas for the development of drugs with high selectivity, low toxicity, good stability and their clinical application in the near future.

## References

[B1] Ali I. Gulfam M. Jo S. H. Seo J. W. Rizwan A. Park S. H. (2022). Reduction-responsive and bioorthogonal carboxymethyl cellulose based soft hydrogels cross-linked via IEDDA click chemistry for cancer therapy application. Int. J. Biol. Macromol. 219, 109–120. 10.1016/j.ijbiomac.2022.07.229 35931291

[B2] Aljohani F. S. Rezki N. Aouad M. R. Elwakil B. H. Hagar M. Sheta E. (2022). Synthesis, characterization and nanoformulation of novel sulfonamide-1,2,3-triazole molecular conjugates as potent antiparasitic agents. Int. J. Mol. Sci. 23 (8), 4241. 10.3390/ijms23084241 35457059 PMC9025934

[B3] Ashe K. (2022). Chemistry just a click away. Nat. Chem. 14 (12), 1341. 10.1038/s41557-022-01108-7 36517563

[B4] Basurto I. M. Passipieri J. A. Gardner G. M. Smith K. K. Amacher A. R. Hansrisuk A. I. (2022). Photoreactive hydrogel stiffness influences volumetric muscle loss repair. Tissue Eng. Part A 28 (7-8), 312–329. 10.1089/ten.TEA.2021.0137 34409861 PMC9057873

[B5] Bhattacherjee D. Kovalev I. S. Kopchuk D. S. Rahman M. Santra S. Zyryanov G. V. (2022). Mechanochemical approach towards multi-functionalized 1,2,3-triazoles and anti-seizure drug rufinamide analogs using copper beads. Molecules 27 (22), 7784. 10.3390/molecules27227784 36431885 PMC9693609

[B6] Bonardd S. Maiti B. Grijalvo S. Rodriguez J. Enshaei H. Kortaberria G. (2022). Biomass-derived isosorbide-based thermoresponsive hydrogel for drug delivery. Soft Matter 18 (26), 4963–4972. 10.1039/d2sm00623e 35748523

[B7] Brauer J. Mötzing M. Gröst C. Hoffmann R. Berg T. (2022). Templated generation of a bcl-xL inhibitor by isomer-free SPAAC based on azacyclonon-5-yne. Chemistry 28 (66), e202202259. 10.1002/chem.202202259 35989238 PMC9827882

[B8] Brown T. Cao M. Zheng Y. G. (2022). Synthesis and activity of triazole-adenosine analogs as protein arginine methyltransferase 5 inhibitors. Molecules 27 (12), 3779. 10.3390/molecules27123779 35744905 PMC9228412

[B9] Cao X. T. Ngan Tran T. Q. Ngo D. H. Tai D. C. Kumar S. (2022). Click-chemistry-mediated synthesis of silver nanoparticle-supported polymer-wrapped carbon nanotubes: Glucose sensor and antibacterial material. ACS Omega 7 (42), 37095–37102. 10.1021/acsomega.2c02832 36312403 PMC9609054

[B10] Chang C. Y. Johnson H. C. Babb O. Fishel M. L. Lin C. C. (2021). Biomimetic stiffening of cell-laden hydrogels via sequential thiol-ene and hydrazone click reactions. Acta Biomater. 130, 161–171. 10.1016/j.actbio.2021.05.054 34087443 PMC8316407

[B11] Cheshmazar N. Hemmati S. Hamzeh-Mivehroud M. Sokouti B. Zessin M. Schutkowski M. (2022). Development of new inhibitors of HDAC1-3 enzymes aided by in silico design strategies. J. Chem. Inf. Model 62 (10), 2387–2397. 10.1021/acs.jcim.1c01557 35467871

[B12] Daher S. S. Lee M. Jin X. Teijaro C. N. Barnett P. R. Freundlich J. S. (2022). Alternative approaches utilizing click chemistry to develop next-generation analogs of solithromycin. Eur. J. Med. Chem. 233, 114213. 10.1016/j.ejmech.2022.114213 35240514 PMC9009214

[B13] Dhameja M. Kumar H. Kurella S. Uma A. Gupta P. (2022). Flavone-1,2,3-triazole derivatives as potential α-glucosidase inhibitors: Synthesis, enzyme inhibition, kinetic analysis and molecular docking study. Bioorg Chem. 127, 106028. 10.1016/j.bioorg.2022.106028 35868105

[B14] Dong J. Varbanov M. Philippot S. Vreken F. Zeng W. B. Blay V. (2023). Ligand-based discovery of coronavirus main protease inhibitors using MACAW molecular embeddings. J. Enzyme Inhib. Med. Chem. 38 (1), 24–35. 10.1080/14756366.2022.2132486 36305272 PMC9621234

[B15] Dwivedi A. R. Rawat S. S. Kumar V. Kumar N. Anand P. Yadav R. P. (2022). Synthesis and screening of novel 4-N-heterocyclic-2-aryl-6,7,8-trimethoxyquinazolines as antiproliferative and tubulin polymerization inhibitors. Bioorg Med. Chem. 72, 116976. 10.1016/j.bmc.2022.116976 36067627

[B16] Elganzory H. H. Alminderej F. M. El-Bayaa M. N. Awad H. M. Nossier E. S. El-Sayed W. A. (2022). Design, synthesis, anticancer activity and molecular docking of new 1,2,3-triazole-based glycosides bearing 1,3,4-thiadiazolyl, indolyl and arylacetamide scaffolds. Molecules 27 (20), 6960. 10.3390/molecules27206960 36296551 PMC9611297

[B17] Guan Q. Zhou L. L. Zhou W. Dong Y. B. (2022). A vinyl-decorated covalent organic framework for ferroptotic cancer therapy via visible-light-triggered cysteine depletion. J. Mater Chem. B 10 (43), 8894–8909. 10.1039/d2tb01815b 36260007

[B18] Guindani C. Dozoretz P. Araújo P. H. H. Ferreira S. R. S. de Oliveira D. (2019). N-acetylcysteine side-chain functionalization of poly(globalide-co-ε-caprolactone) through thiol-ene reaction. Mater Sci. Eng. C Mater Biol. Appl. 94, 477–483. 10.1016/j.msec.2018.09.060 30423732

[B19] Hashad R. A. Singla R. Kaur Bhangu S. Jap E. Zhu H. Peleg A. Y. (2022). Chemoenzymatic surface decoration of Nisin-shelled nanoemulsions: Novel targeted drug-nanocarriers for cancer applications. Ultrason. Sonochem 90, 106183. 10.1016/j.ultsonch.2022.106183 36201933 PMC9554623

[B20] Hou G. Qian J. Xu W. Sun T. Wang Y. Wang J. (2019). A novel pH-sensitive targeting polysaccharide-gold nanorod conjugate for combined photothermal-chemotherapy of breast cancer. Carbohydr. Polym. 212, 334–344. 10.1016/j.carbpol.2019.02.045 30832865

[B21] Jang K. J. Lee W. S. Park S. Han J. Kim J. E. Kim B. M. (2021). Sulfur(VI) fluoride exchange (SuFEx)-Mediated synthesis of the chitosan-PEG conjugate and its supramolecular hydrogels for protein delivery. Nanomater. (Basel) 11 (2), 318. 10.3390/nano11020318 PMC7912644 33513757

[B22] Kutkat O. Kandeil A. Moatasim Y. Elshaier Y. A. M. M. El-Sayed W. A. Gaballah S. T. (2022). *In vitro* and *in vivo* antiviral studies of new heteroannulated 1,2,3-triazole glycosides targeting the neuraminidase of influenza A viruses. Pharm. (Basel) 15 (3), 351. 10.3390/ph15030351 PMC8950700 35337148

[B23] Liao J. Peng H. Liu C. Li D. Yin Y. Lu B. (2021). Dual pH-responsive-charge-reversal micelle platform for enhanced anticancer therapy. Mater Sci. Eng. C Mater Biol. Appl. 118, 111527. 10.1016/j.msec.2020.111527 33255080

[B24] Macedo Vaz S. de Freitas Silva M. Dos Reis Rosa Franco G. Jorge R. Guimaraes M. Motta R. da Silva F. Goncalves Castro N. (2022). Synthesis and biological evaluation of 4-hydroxy-methylpiperidinyl-N-benzyl-acylarylhydrazone hybrids designed as novel multifunctional drug candidates for Alzheimer's disease. Bioorg Med. Chem. 71, 116952. 10.1016/j.bmc.2022.116952 35930852

[B25] Maslov I. O. Zinevich T. V. Kirichenko O. G. Trukhan M. V. Shorshnev S. V. Tuaeva N. O. (2022). Design, synthesis and biological evaluation of neogliptin, a novel 2-azabicyclo[2.2.1]heptane-based inhibitor of dipeptidyl peptidase-4 (DPP-4). Pharm. (Basel) 15 (3), 273. 10.3390/ph15030273 PMC8949241 35337071

[B26] Mekonnen Sanka B. Mamo Tadesse D. Teju Bedada E. Mengesha E. T. Babu G. N. (2022). Design, synthesis, biological screening and molecular docking studies of novel multifunctional 1,4-di (aryl/heteroaryl) substituted piperazine derivatives as potential antitubercular and antimicrobial agents. Bioorg Chem. 119, 105568. 10.1016/j.bioorg.2021.105568 34968884

[B27] Mironov M. E. Rybalova T. V. Pokrovskii M. A. Emaminia F. Gandalipov E. R. Pokrovskii A. J. (2023). Synthesis of fully functionalized spirostanic 1,2,3-triazoles by the three component reaction of diosgenin azides with acetophenones and aryl aldehydes and their biological evaluation as antiproliferative agents. Steroids 190, 109133. 10.1016/j.steroids.2022.109133 36328088

[B28] Mohammed H. H. H. Abd El-Hafeez A. A. Ebeid K. Mekkawy A. I. Abourehab M. A. S. Wafa E. I. (2022). New 1,2,3-triazole linked ciprofloxacin-chalcones induce DNA damage by inhibiting human topoisomerase I& II and tubulin polymerization. J. Enzyme Inhib. Med. Chem. 37 (1), 1346–1363. 10.1080/14756366.2022.2072308 35548854 PMC9116245

[B29] Mokariya J. A. Kalola A. G. Prasad P. Patel M. P. (2022). Simultaneous ultrasound- and microwave-assisted one-pot 'click' synthesis of 3-formyl-indole clubbed 1,2,3-triazole derivatives and their biological evaluation. Mol. Divers 26 (2), 963–979. 10.1007/s11030-021-10212-8 33834361

[B30] Nakajima M. Adachi Y. Nemoto T. (2022). Computation-guided asymmetric total syntheses of resveratrol dimers [published correction appears in Nat Commun. 2022 Apr 27;13(1):2418. Nat. Commun. 13 (1), 152. 10.1038/s41467-021-27546-4 35013143 PMC8748746

[B31] Nsira A. Mtiraoui H. Chniti S. Al-Ghulikah H. Gharbi R. Msaddek M. (2022). Regioselective one-pot synthesis, biological activity and molecular docking studies of novel conjugates N-(p-Aryltriazolyl)-1,5-benzodiazepin-2-ones as potent antibacterial and antifungal agents. Molecules 27 (13), 4015. 10.3390/molecules27134015 35807263 PMC9268147

[B32] Oekchuae S. Sirirak J. Charoensuksai P. Wongprayoon P. Chuaypen N. Boonsombat J. (2022). The design and synthesis of a new series of 1,2,3-triazole-cored structures tethering aryl urea and their highly selective cytotoxicity toward HepG2. Pharm. (Basel) 15 (5), 504. 10.3390/ph15050504 PMC9147274 35631331

[B33] Oliveira A. Moura S. Pimentel L. Neto J. Dantas R. Silva-Jr F. (2022). New imatinib derivatives with antiproliferative activity against A549 and K562 cancer cells. Molecules 27 (3), 750. 10.3390/molecules27030750 35164014 PMC8838532

[B34] ópez S. Gracia I. Plaza-Pedroche R. Rodriguez J. F. Perez-Ortiz J. M. Rodriguez-Lopez J. (2022). *In vitro* antioxidant and pancreatic anticancer activity of novel 5-fluorouracil-coumarin conjugates. Pharmaceutics 14 (10), 2152. 10.3390/pharmaceutics14102152 36297585 PMC9607493

[B35] Pansare A. V. Pansare P. V. Shedge A. A. Pansare S. V. Patil V. R. Terrasi G. P. (2022). Click gold quantum dots biosynthesis with conjugation of quercetin for adenocarcinoma exertion. RSC Adv. 12 (29), 18425–18430. 10.1039/d2ra02529a 35799927 PMC9218964

[B36] Qayed W. S. Hassan M. A. El-Sayed W. M. Rogério A Silva J. Aboul-Fadl T. (2022). Novel azine linked hybrids of 2-indolinone and thiazolodinone scaffolds as CDK2 inhibitors with potential anticancer activity: *In silico* design, synthesis, biological, molecular dynamics and binding free energy studies. Bioorg Chem. 126, 105884. 10.1016/j.bioorg.2022.105884 35623140

[B37] Sousa G. F. Afewerki S. Dittz D. Santos F. E. P. Gontijo D. O. Scalzo S. R. A. (2022). Catalyst-free click chemistry for engineering chondroitin sulfate-multiarmed PEG hydrogels for skin tissue engineering. J. Funct. Biomater. 13 (2), 45. 10.3390/jfb13020045 35466227 PMC9036249

[B38] Spieler V. Ludwig M. G. Dawson J. Tigani B. Littlewood-Evans A. Safina C. (2020). Targeting interleukin-4 to the arthritic joint. J. Control Release 326, 172–180. 10.1016/j.jconrel.2020.07.005 32653504

[B39] Syam Y. M. Anwar M. M. Abd El-Karim S. S. Elokely K. M. Abdelwahed S. H. (2022). New quinoxaline-based derivatives as PARP-1 inhibitors: Design, synthesis, antiproliferative, and computational studies. Molecules 27 (15), 4924. 10.3390/molecules27154924 35956876 PMC9370283

[B40] Taghour M. S. Elkady H. Eldehna W. M. El-Deeb N. Kenawy A. M. Elkaeed E. B. (2022). Design, synthesis, anti-proliferative evaluation, docking, and MD simulations studies of new thiazolidine-2,4-diones targeting VEGFR-2 and apoptosis pathway. PLoS One 17 (9), e0272362. 10.1371/journal.pone.0272362 36149902 PMC9506633

[B41] Tatarinov D. A. Garifullin B. F. Belenok M. G. Andreeva O. V. Strobykina I. Y. Shepelina A. V. (2022). The first 5'-phosphorylated 1,2,3-triazolyl nucleoside analogues with uracil and quinazoline-2,4-dione moieties: A synthesis and antiviral evaluation. Molecules 27 (19), 6214. 10.3390/molecules27196214 36234748 PMC9573387

[B42] Wadanambi P. M. Jayathilaka N. Seneviratne K. N. (2023). A computational study of carbazole alkaloids from Murraya koenigii as potential SARS-CoV-2 main protease inhibitors. Appl. Biochem. Biotechnol. 195 (1), 573–596. 10.1007/s12010-022-04138-6 36107386 PMC9474281

[B43] Wallat J. D. Harrison J. K. Pokorski J. K. (2018). pH responsive doxorubicin delivery by fluorous polymers for cancer treatment. Mol. Pharm. 15 (8), 2954–2962. 10.1021/acs.molpharmaceut.7b01046 29381366

[B44] Wang P. Li M. Zhou F. Yang Y. Yin X. Zhang X. B. (2022). COF-based nanoreactors for click-activated prodrug delivery and precise anti-vascular therapy. Chem. Commun. (Camb). 58 (79), 11107–11110. 10.1039/d2cc03931a 36102676

[B45] Wu F. Zhou Y. Li L. Shen X. Chen G. Wang X. (2020). Computational approaches in preclinical studies on drug discovery and development. Front. Chem. 8, 726. 10.3389/fchem.2020.00726 33062633 PMC7517894

[B46] Xu G. Bai X. Dang Q. (2020). Aromatic heterocycles as productive dienophiles in the inverse electron-demand diels-alder reactions of 1,3,5-triazines. Acc. Chem. Res. 53 (4), 773–781. 10.1021/acs.accounts.9b00604 32227911

[B47] Yun W. S. Shim M. K. Lim S. Song S. Kim J. Yang S. (2022). Mesenchymal stem cell-mediated deep tumor delivery of gold nanorod for photothermal therapy. Nanomater. (Basel). 12 (19), 3410. 10.3390/nano12193410 PMC9565344 36234538

[B48] Zhang F. G. Chen Z. Tang X. Ma J. A. (2021). Triazines: Syntheses and inverse electron-demand diels-alder reactions. Chem. Rev. 121 (23), 14555–14593. 10.1021/acs.chemrev.1c00611 34586777

[B49] Zhang X. Zhang S. Zhao S. Wang X. Liu B. Xu H. (2021). Click chemistry in natural product modification. Front. Chem. 9, 774977. 10.3389/fchem.2021.774977 34869223 PMC8635925

[B50] Zhang Z. Jamieson C. S. Zhao Y. L. Li D. Ohashi M. Houk K. N. (2019). Enzyme-catalyzed inverse-electron demand diels–alder reaction in the biosynthesis of antifungal Ilicicolin H. J. Am. Chem. Soc. 141 (14), 5659–5663. 10.1021/jacs.9b02204 30905148 PMC6585442

[B51] Zhou M. Wang W. Wang Z. Wang Y. Zhu Y. Lin Z. (2022). Discovery and computational studies of 2-phenyl-benzoxazole acetamide derivatives as promising P2Y14R antagonists with anti-gout potential. Eur. J. Med. Chem. 227, 113933. 10.1016/j.ejmech.2021.113933 34689072

